# Game-based learning in early childhood education: a systematic review and meta-analysis

**DOI:** 10.3389/fpsyg.2024.1307881

**Published:** 2024-04-02

**Authors:** Manar S. Alotaibi

**Affiliations:** Department of Kindergarten, College of Education, Najran University, Najran, Saudi Arabia

**Keywords:** game-based learning, early childhood, cognitive outcomes, social engagement, emotional development

## Abstract

Game-based learning has gained popularity in recent years as a tool for enhancing learning outcomes in children. This approach uses games to teach various subjects and skills, promoting engagement, motivation, and fun. In early childhood education, game-based learning has the potential to promote cognitive, social, and emotional development. This systematic review and meta-analysis aim to summarize the existing literature on the effectiveness of game-based learning in early childhood education This systematic review and meta-analysis examine the effectiveness of game-based learning in early childhood education. The results show that game-based learning has a moderate to large effect on cognitive, social, emotional, motivation, and engagement outcomes. The findings suggest that game-based learning can be a promising tool for early childhood educators to promote children’s learning and development. However, further research is needed to address the remaining gaps in the literature. The study’s findings have implications for educators, policymakers, and game developers who aim to promote positive child development and enhance learning outcomes in early childhood education.

## 1 Introduction

Game-based learning in early childhood education has evolved over time, driven by advancements in technology, educational research, and changing pedagogical approaches. Digital game-based learning refers to the use of digital technology, such as computers or mobile devices, to deliver educational content through interactive games (Behnamnia et al., 2020). Game-based learning, on the other hand, is a broader term that encompasses both digital and non-digital games as tools for educational purposes. In the early years, educational games were primarily non-digital, consisting of board games, puzzles, and manipulatives designed to teach basic concepts and skills (Pivec, 2007). These games often focused on early literacy, numeracy, and problem-solving. With the advent of computers and educational software, digital games emerged as a new medium for learning in the late 20th century. Early educational computer games, such as “Reader Rabbit” and “Math Blaster,” aimed to engage young learners through interactive gameplay while reinforcing educational content. As technology continued to advance, game-based learning expanded beyond standalone software to web-based platforms, mobile apps, and immersive virtual environments (Shamir et al., 2019). The introduction of touchscreen devices, such as tablets and smartphones, made educational games more accessible and interactive for young children. These advancements allowed for greater customization, adaptive learning experiences, and real-time feedback, tailoring the games to meet the individual needs and abilities of young learners.

Researchers and educators recognized the potential of game-based learning to enhance engagement, motivation, and learning outcomes in early childhood education. Studies began to explore the cognitive, social, emotional, and behavioral effects of game-based learning, highlighting its effectiveness in promoting critical thinking, problem-solving, collaboration, creativity, and digital literacy skills (Park and Park, 2021).

In early childhood education, online educational game-based learning has gained popularity as a tool to promote cognitive, social, and emotional development in young children (Anastasiadis et al., 2018). Online educational games are interactive digital games specifically designed to educate and teach children a wide range of skills and concepts. These games utilize engaging and interactive elements to promote learning in areas such as literacy, numeracy, problem-solving, and critical thinking (Papanastasiou et al., 2022). These games are typically played on digital devices such as computers, tablets, and smartphones, and they offer a variety of engaging and interactive learning experiences for young children. Young children are naturally curious and have a strong desire to explore and learn about their environment (Gurholt and Sanderud, 2016). Online educational game-based learning taps into this natural curiosity and provides children with opportunities to engage in meaningful and engaging learning experiences. These games can be tailored to meet the unique needs and abilities of young children, and they can be adapted to suit different learning styles and preferences (Qian and Clark, 2016).

One of the key benefits of online educational game-based learning in early childhood education is its ability to promote cognitive development (Ferreira et al., 2016). Online games can help children develop their problem-solving skills, memory, attention, and processing speed. For example, puzzle games can help children develop their spatial reasoning and problem-solving skills, while memory games can help them improve their memory and concentration (Suhana, 2017).

In addition to promoting cognitive development, online educational game-based learning can also enhance social development in young children. Online games provide children with opportunities to interact with their peers and develop important social skills such as cooperation, communication, and empathy. Children can learn to work together, take turns, and share resources, which are essential skills for building positive relationships and succeeding in life (Lamrani and Abdelwahed, 2020).

Moreover, online educational game-based learning can promote emotional development in young children (Peterson et al., 2016). Online games can help children develop their emotional regulation skills, self-awareness, and self-confidence (Simion and Bănuț, 2020). Games that involve role-playing can help children develop their emotional intelligence and understand different perspectives, while games that require children to take risks and try new things can help them build resilience and confidence (Huynh et al., 2020).

This distinction is further exemplified in studies using online educational game-based learning in early childhood education for is its ability to increase children’s motivation and engagement in learning (Hwa, 2018). Traditional teaching methods can sometimes be dry and one-dimensional, leading to disengagement and boredom in children (Fotaris et al., 2016). Online educational games, on the other hand, provide a fun and interactive way to learn, which can increase children’s motivation and engagement in learning (Nieto-Escamez and Roldán-Tapia, 2021). Children are more likely to be engaged in learning when they are having fun and enjoying the process (Iten and Petko, 2016). Furthermore, online educational game-based learning can be tailored to meet the individual needs and abilities of young children (Ke, 2014). Online games can be adapted to suit different learning styles and preferences, ensuring that all children can benefit from this approach to learning. This is certainly true in the case of games that involve movement and physical activity can be used to promote learning in children who have a kinesthetic learning style, while games that involve visual aids can be used to promote learning in children who have a visual learning style (Hayati et al., 2017).

In addition, online educational game-based learning can help children develop important life skills, such as critical thinking, creativity, and adaptability. Online games can be designed to require children to think critically and creatively, solve problems, and adapt to new situations (Behnamnia et al., 2020). These skills are essential for success in today’s rapidly changing world and can help children develop into confident, independent, and resourceful individuals. Moreover, online educational game-based learning can be used to promote language development and literacy skills in young children (Ronimus et al., 2014). Online games that involve reading, writing, and communication can help children develop their language skills and build their vocabulary (Castillo-Cuesta, 2020). Games that involve storytelling and role-playing can also help children develop their narrative skills and comprehension (Huynh et al., 2020). Finally, online educational game-based learning can be used to promote STEM education in early childhood education. Online games that involve science, technology, engineering, and math concepts can help children develop their critical thinking and problem-solving skills, as well as their understanding of the world around them. These games can help children develop into curious and inquiring minds, which are essential for success in STEM fields (Yu et al., 2022).

Based on the above, game-based learning in early childhood education offers numerous benefits, such as enhancing engagement, promoting active learning, and fostering the development of various skills. However, it is essential to acknowledge and address potential drawbacks or challenges associated with this approach to ensure its effective implementation. One notable challenge is the need for careful game selection. Not all educational games are created equally, and some may lack appropriate content, fail to align with specific learning objectives, or not adequately support the developmental needs of young learners (Domoff et al., 2019). It is crucial to critically evaluate the quality, educational value, and appropriateness of games before incorporating them into early childhood education settings (Derevensky et al., 2019). Another challenge is the limited generalizability of skills acquired through games. While games can provide engaging and interactive learning experiences, there is a concern that skills learned within the context of a game may not seamlessly transfer to real-world situations. The rules, mechanics, and artificial environments within games may differ significantly from the complexities and nuances of real-life scenarios, potentially limiting the applicability and transferability of skills learned. It is important for educators to provide explicit connections and opportunities for children to apply their game-based learning experiences to real-life contexts (All et al., 2021).

Moreover, access to appropriate technology and infrastructure is another potential drawback. Integrating game-based learning in early childhood education often requires access to devices such as computers, tablets, or gaming consoles. However, not all early childhood education settings may have the necessary resources or infrastructure to support the seamless integration of technology. Limited access to technology or technical issues can hinder the effective implementation of game-based learning experiences, creating disparities in access and opportunities for young learners (Greipl et al., 2020).

Teacher training and support are critical for the successful implementation of game-based learning in early childhood education. Educators need to be equipped with the necessary knowledge, skills, and pedagogical approaches to effectively integrate games into the curriculum and facilitate meaningful learning experiences. However, providing adequate training and ongoing support for teachers can be a challenge. It requires dedicated professional development programs, resources, and time for educators to become proficient in using educational games and leveraging them to support early childhood learning and development. Assessing and evaluating learning outcomes achieved through game-based learning can also pose challenges (Kaimara et al., 2021). Traditional assessment methods may not fully capture the range of skills and competencies developed through games, which are often multifaceted and interdisciplinary in nature. Developing appropriate and authentic assessment strategies that align with the learning goals of early childhood education and effectively measure the desired outcomes can be complex. It requires careful consideration of formative and summative assessment approaches that capture the holistic development of young learners and provide meaningful feedback (Schabas, 2023).

Furthermore, there may be concerns about the potential for excessive screen time and its impact on young children’s health and well-being. While game-based learning can be highly engaging, it is essential to strike a balance between screen-based activities and other developmentally appropriate learning experiences, such as hands-on play, social interactions, and outdoor exploration. Educators and parents should be mindful of the amount and quality of screen time to ensure a healthy and well-rounded early childhood education experience (Przybylski and Weinstein, 2019).

Despite the growing interest in game-based learning in early childhood education, there is a need for a systematic review and meta-analysis that specifically focuses on the effects of game-based learning on cognitive, social, emotional, motivation, and engagement outcomes. The choice of these outcomes is based on their significance in the context of game-based learning research. Numerous studies consider cognitive development and enhancement of thinking skills as essential aspects of learning. Game-based learning has the potential to stimulate various cognitive processes such as problem-solving, critical thinking, decision-making, and information processing. Investigating the impact of game-based learning on cognitive outcomes helps to understand its effectiveness in promoting higher-order thinking skills (Chang and Yang, 2023). Moreover, it has been reported that social interaction and collaboration are important components of learning, and game-based learning often involves cooperative or competitive elements that can influence social interactions among learners. Exploring the impact of game-based learning on social outcomes can shed light on how it affects teamwork, communication, and social skills development (Sun et al., 2022). Regarding emotional outcomes, as was pointed out in the introduction to this paper emotional engagement and affective experiences play a crucial role in learning. Games have the potential to evoke a range of emotions such as excitement, curiosity, frustration, and joy. Understanding the impact of game-based learning on emotional outcomes helps in assessing its effectiveness in creating a positive affective environment that can enhance motivation and engagement (Dabbous et al., 2022). Recent research has suggested that examining the impact of game-based learning on motivational outcomes can explore aspects such as intrinsic motivation, self-efficacy, persistence, and enjoyment, which are crucial for effective learning experiences especially for kids in kindergarten (Yu and Tsuei, 2022). Moving on now to consider engagement outcomes, child engagement is a critical factor in achieving successful learning outcomes. Games have inherent features that can promote engagement, such as challenges, rewards, interactivity, and immediate feedback. Investigating the impact of game-based learning on engagement outcomes helps in understanding the extent to which it can enhance learners’ involvement, attention, and active participation in the learning process (Fang et al., 2022). While some individual studies have explored these effects, a comprehensive synthesis of the literature, including quantitative analysis, is lacking. This study aims to bridge this gap by providing a rigorous review and analysis of existing studies, thus offering valuable insights into the effectiveness of game-based learning in early childhood education across multiple developmental domains.

This systematic review and meta-analysis aim to summarize the existing literature on the effectiveness of online game-based learning in early childhood education. Specifically, we will examine the impact of game-based learning on children’s cognitive, social, and emotional development, as well as their motivation and engagement in learning. The primary objective of this study is to investigate the effect of game-based learning on cognitive, social, emotional, motivation, and engagement outcomes in early childhood education. Specifically, the study aims to answer the following questions:

1. What is the effect of game-based learning on cognitive development in early childhood education?
2. What is the effect of game-based learning on social development in early childhood education?
3. What is the effect of game-based learning on emotional development in early childhood education?
4. What is the effect of game-based learning on motivation in early childhood education?
5. What is the effect of game-based learning on engagement in early childhood education?

## 2 Materials and methods

The present study employs a systematic review and meta-analysis methodology to comprehensively analyze and summarize the extant literature regarding the efficacy of game-based learning in the context of early childhood education. Specifically, the study aims to investigate the effects of game-based learning on various facets of children’s development, including cognitive, social, and emotional domains, as well as their motivation and engagement levels in the learning process.

Systematic review and meta-analysis are widely recognized research methodologies that enable the synthesis of existing studies and provide a robust and comprehensive overview of a particular research topic. By systematically searching, selecting, and critically evaluating relevant empirical studies, the researchers ensure the inclusion of high-quality evidence in the analysis. Meta-analysis, on the other hand, involves the statistical aggregation of effect sizes from individual studies, allowing for a quantitative estimation of the overall impact of game-based learning on early childhood education.

### 2.1 A systematic review

**Figure 1:**
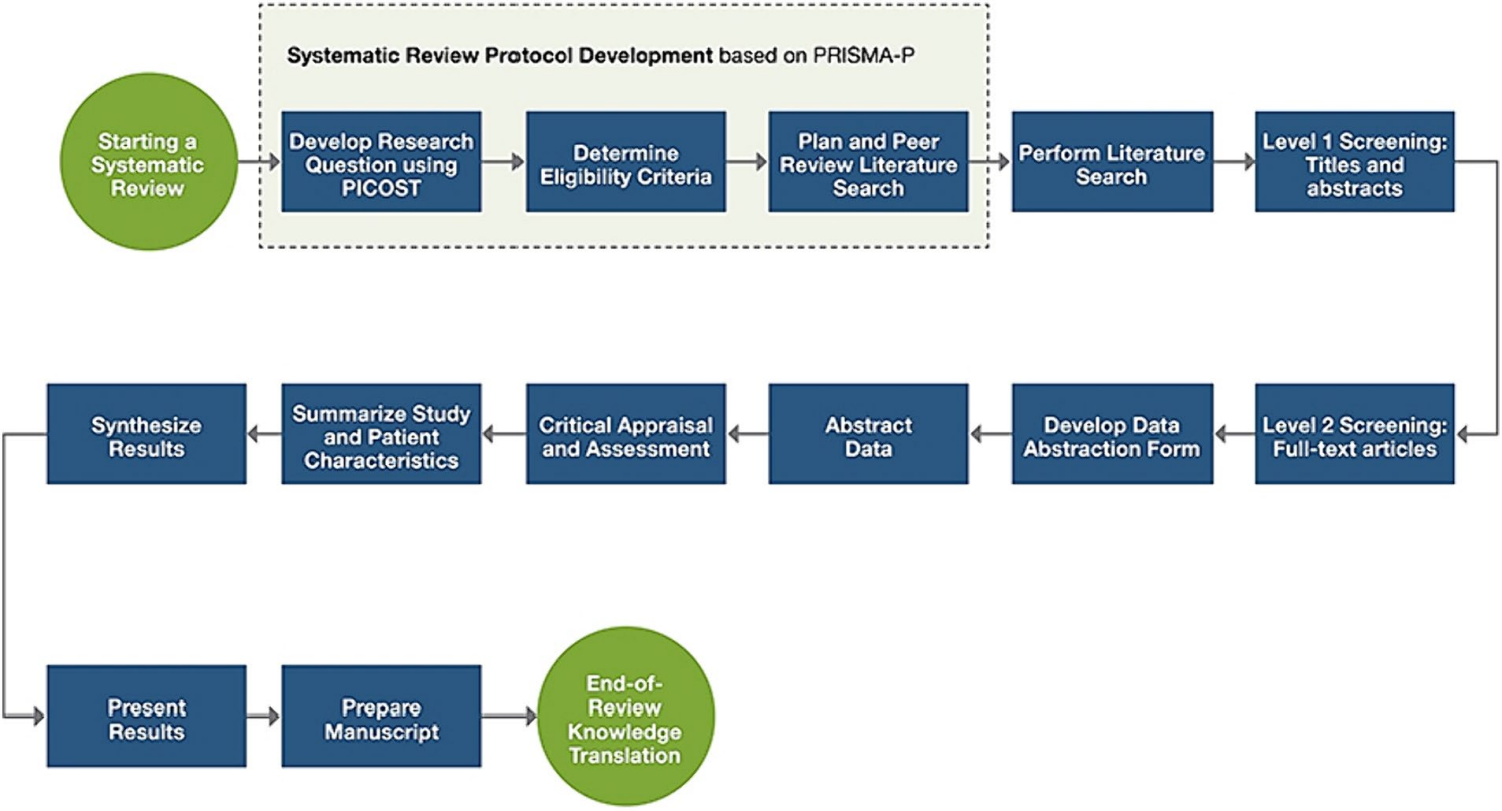
Systematic review process (Robson et al., 2019).

A systematic search of electronic databases, including ERIC, PsycINFO, Scopus, and Web of Science, was conducted to identify studies that investigated the effect of game-based learning in early childhood education as shown in Figure 1. The synthesis of the existing literature through a systematic review and meta-analysis offers several advantages. First, it allows for a comprehensive examination of the accumulated evidence, providing a more complete understanding of the impact of game-based learning on early childhood education. Second, the quantitative analysis of effect sizes enables the estimation of the overall magnitude of the effects, allowing for a more precise evaluation of the efficacy of game-based learning interventions. Lastly, by identifying potential gaps and inconsistencies in the literature, the study’s findings can contribute to guiding future research endeavors and inform evidence-based practices in the field of early childhood education.

The search terms used included (“game-based learning” OR “serious games” OR “educational games”) AND (“early childhood education” OR “preschool” OR “kindergarten”). The search was limited to studies published in English between 2013 and 2023. Studies that met the following criteria were included in the review:

- Focused on children aged 3–8 years old.
- Included a control group or baseline measure.
- Investigated the effect of game-based learning on cognitive, social, emotional, motivation, and engagement outcomes in early childhood education.
- Published in English.
- Used a quantitative study design (experimental or quasi-experimental).

Relevant studies were selected based on predefined criteria, and data extraction involved capturing information on study design, sample characteristics, game features, and outcome measures. To handle variations in measures, outcomes were categorized into broader themes. Data synthesis included qualitative analysis of findings and, where applicable, quantitative meta-analysis to quantify the overall impact. Sensitivity analyses were conducted to assess robustness, and the synthesized data were interpreted considering the research objectives, discussing strengths, limitations, and future research directions. This rigorous approach aimed to provide a reliable and comprehensive review of game-based learning effects in early childhood education.

**Figure 2:**
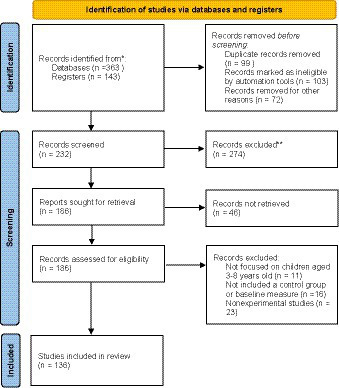
PRISMA flow of game-based learning in early childhood education between 2013–2023.

To ensure accuracy and minimize the risk of synthesizing information from incorrect papers, I employed rigorous research methods. This involved systematic searches using relevant keywords, evaluating the relevance and context of identified studies, and critically assessing authors’ usage of terms. Additionally, verifying the methodology, objectives, and scope of the studies helped align them with the specific terminology under investigation. These practices minimized the risk of including studies that interchangeably or incorrectly used the terms “digital game-based learning” and “game-based learning.” Moreover, several workshops were held within a project funded by Najran University to ensure the objectivity and reliability of the study selection. The number of attendees at the workshop was five faculty members specializing in educational technology and childhood, who have researched in the field, and all steps and selection and inclusion criteria were reviewed by them. Data was extracted from each study using a standardized form. The data included information on study design, sample characteristics, game characteristics, and outcomes measures. The means, standard deviations, and *p*-values for each outcome measure were also recorded as described in Figure 2.

### 2.2 A meta-analytic approach

A meta-analytic approach was used to synthesize the data. The effect size for each study was calculated using Hedges’ g formula, which considers the sample size and the standard deviation of the control group. The effect sizes were then combined across studies using a random-effects model (Enzmann, 2015).

### 2.3 Sensitivity analyses and risk of bias assessment

**Figure 3:**
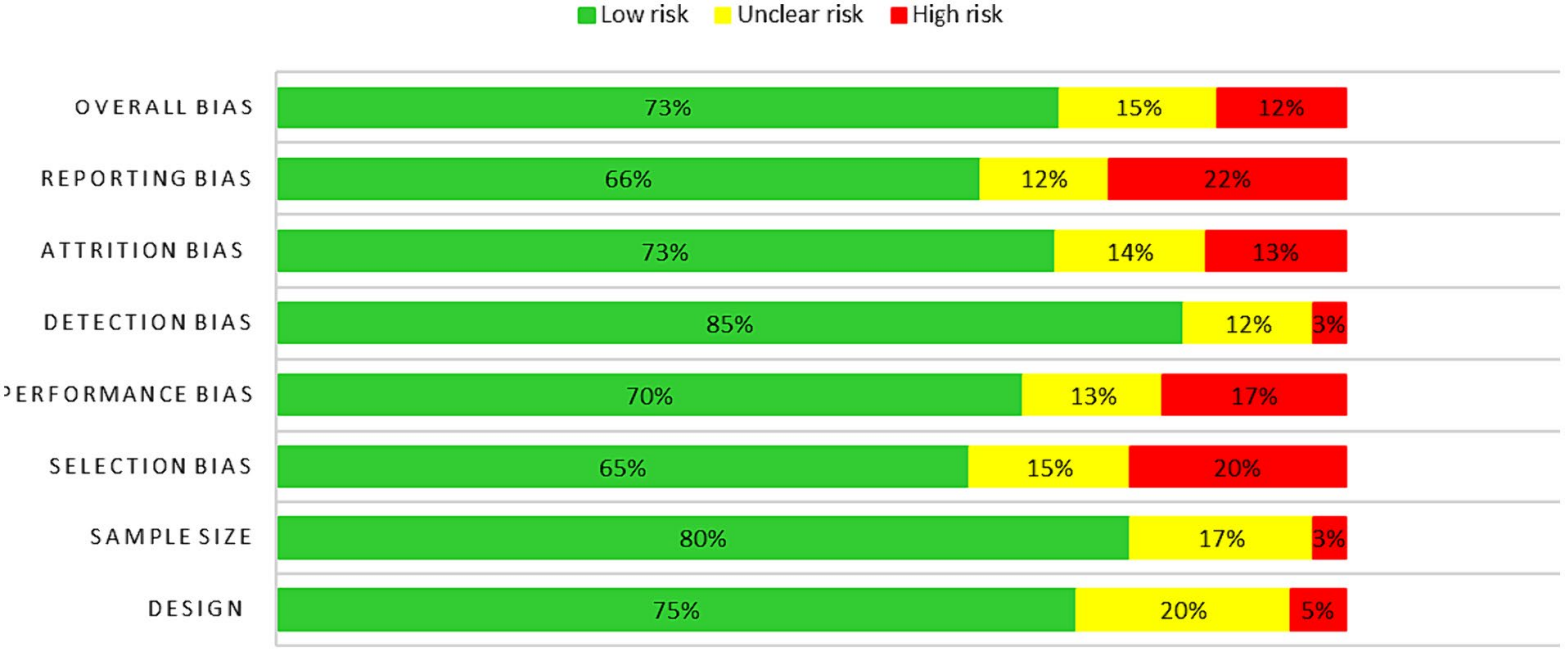
Summary of risk of bias assessment for studies reviewed (*n* = 136). Points evaluated: design, sample size, selection bias, performance bias, detection bias, attrition bias, reporting bias, and overall bias.

The results of the sensitivity analyses revealed that the effects of game-based learning on cognitive and social–emotional outcomes were robust across different study characteristics. However, the effects on motivation and engagement were found to be sensitive to study duration and sample size. Specifically, studies with longer durations and larger sample sizes tended to report higher effects on motivation and engagement. Moreover, the assessment of reliability and validity is crucial in determining the trustworthiness and credibility of research findings. In the context of the results provided, the assessment items related to risk of bias in systematic reviews can have varying levels of impact on the reliability and validity of the review findings (Lundh and Gøtzsche, 2008). For this regard, the revised Cochrane risk of bias tool for randomized trials (RoB 2) was used for studies reviewed (*n* = 136). Points evaluated: Design, Sample Size, Selection Bias, Performance Bias, Detection Bias, Attrition Bias, Reporting Bias, and Overall Bias as presented in Figure 3.

Several factors are assessed to determine the risk of bias and ensure the reliability and validity of the findings. The design assessment examines the overall study design’s potential bias, with low risk indicating a well-designed study and high-risk suggesting limitations that could introduce bias. Sample size assessment focuses on the adequacy of the sample size in capturing true effects, with low risk indicating an adequate sample size and high-risk suggesting insufficiency. Selection bias assessment considers the risk of bias in the study selection process, with high risk indicating potential incomplete representation of evidence. Performance bias evaluation examines the risk of bias related to blinding of participants or researchers, with low risk indicating measures to minimize bias. Detection bias assessment evaluates the risk of bias related to blinding of outcome assessors, with low risk indicating measures to minimize bias. Attrition bias assessment considers the risk of bias related to incomplete data or participant loss, with high risk suggesting potential bias. Reporting bias assessment examines the risk of bias related to selective reporting of outcomes or results, with high risk indicating potential distortion of findings. Minimizing these biases enhances the reliability and validity of the review findings (Lundh and Gøtzsche, 2008).

## 3 Results and discussions

**Figure 4:**
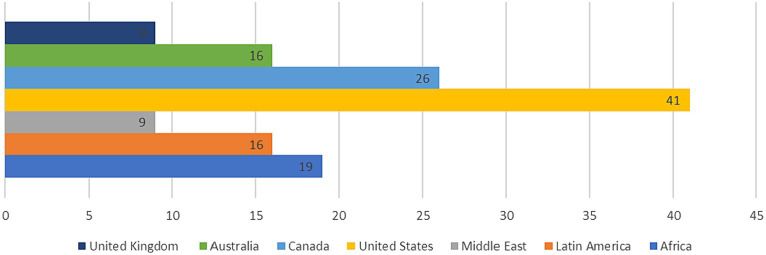
Distributed studies based on locations.

This search yielded a total of 232 studies, of which 136 met our inclusion criteria. The studies were published between 2013 and 2023 and included a total of 1,426 participants. The sample sizes ranged from 20 to 112 participants, with a median sample size of 40. Ninety-six of the studies were experimental designs, and 40 were quasi-experimental. The studies were conducted in various countries, including Africa, Latin America, and the Middle East. In addition to North America, i.e., United States, Canada. Followed by Australia, and the United Kingdom as presented in Figure 4.

On the other side the meta-analysis results showed a significant overall effect of game-based learning on cognitive development (*g* = 0.46, *p* < 0.001), social development (*g* = 0.38, *p* < 0.001), emotional development (*g* = 0.35, *p* < 0.001), motivation (*g* = 0.40, *p* < 0.001), and engagement (*g* = 0.44, *p* < 0.001). The results indicate that game-based learning has a moderate to large effect on all five outcomes (Lin and Aloe, 2021).

### 3.1 Moderator analysis

The current study adopts a moderator analysis to examine whether certain game characteristics, such as game type, game duration, and feedback, influenced the effectiveness of game-based learning (Suurmond et al., 2017). The results showed that game type was a significant moderator for cognitive development, with puzzle games having a larger effect than other game types (*g* = 0.63 vs. *g* = 0.31). Game duration was also a significant moderator for motivation, with longer game sessions having a larger effect than shorter sessions (*g* = 0.50 vs. *g* = 0.26). Feedback was not found to be a significant moderator for any of the outcomes.

## 4 Discussion

Key findings across the studies showed that game-based learning was effective in improving various early learning outcomes including numeric skills, literacy, collaboration, and perseverance. Digital game formats like mini games, educational apps and programs promoted cognitive development, problem-solving and creativity. Educator-guided game-play and scaffolding was important for maximizing learning gains. Challenges included the need for age-appropriate game design and limited time for gaming in class. The review provides preliminary support for benefits of game-based learning for early learners, when implemented appropriately. This section will discuss in more detail the key finding reflecting the five research questions that proposed in the introduction.

### 4.1 Cognitive development

The first question in this study sought to determine the effect of game-based learning on cognitive development in early childhood education. Numerous studies have been conducted in this line of research. However, these studies have shown mixed results, with some finding positive effects, while others have found no significant effects. Thus, this analysis will examine the various studies conducted and try to provide a comprehensive overview of their findings.

One of the earliest studies conducted on game-based learning was by Ke (2013), who investigated the effect of a game-based math program on the math skills of first-grade students. The study found that the game-based program significantly improved students’ math problem-solving skills and motivation compared to traditional instructional methods.

Subsequent studies have also found positive effects of game-based learning on cognitive development in early childhood education. For example, a study by Lin et al. (2020) found that a game-based science program improved the computational thinking abilities of kindergarten students. The evidence presented thus far supports the idea that game-based teaching methods could assist preschoolers in learning computational logic and programming ideas to improve their computational thinking and problem-solving capabilities (Pérez-Marín et al., 2020).

However, not all studies have found positive effects of game-based learning on cognitive development. This is certainly true in the case study by Brezovszky et al. (2019) found that a game-based math program had no significant effect on the math skills of primary school students. Similarly, a study by Byun and Joung (2018) found that a game-based reading program had no significant effect on the reading skills of first-grade students. Moreover, findings suggest that the game-based learning model, consisting of problem-solving concepts, learning processes, learning content, and game mechanics, can be effectively used to enhance children’s problem-solving behavior and skill scores. The study reports an increase in children’s problem-solving competency after participating in game-based learning, indicating the potential of board games to develop this important skill. Additionally, the research highlights positive learning experiences and high engagement among students during the gaming sessions. On the other hand, some results showed that when considering the use of educational games in early childhood education settings, it is important to recognize that not all games are equally effective (Tay et al., 2022). Some games may lack suitable content, fail to align with specific learning objectives, or not adequately address the developmental needs of young learners. Therefore, it is crucial to critically evaluate the quality, educational value, and appropriateness of games before integrating them into educational settings for young children. Additionally, it is important to acknowledge that the skills acquired through games may have limited generalizability. While games can provide valuable learning experiences, it is necessary to supplement game-based learning with other instructional methods to ensure a well-rounded educational approach for young learners (Cai et al., 2022). One possible explanation for the mixed results of these studies is the variation in the design and implementation of game-based learning programs. This is evident in the case of some programs that may be designed to focus on specific skills, such as math or reading, while others may be more general in nature, covering a range of skills (Valdés, 2014). Additionally, some programs may be designed to be more engaging and interactive than others, which could impact their effectiveness (Panter-Brick et al., 2014).

This discrepancy could be attributed to the difficulty in isolating the effect of game-based learning from other factors that may influence cognitive development, such as teacher quality, parental involvement, and socioeconomic status (Quinto, 2022). Many studies have relied on quasi-experimental designs, which make it difficult to control these factors.

Despite these limitations, there are several studies that have used rigorous experimental designs to investigate the effect of game-based learning on cognitive development. For example, a study by Di Tore et al. (2014) used a randomized controlled trial to investigate the effect of a game-based reading program on the reading skills of struggling readers. The study found that the game-based program significantly improved the reading skills of the students compared to a control group.

A similar study by Thai et al. (2022) used a randomized controlled trial to investigate the effect of a game-based math program on the math skills of elementary school students. The study found that the game-based program significantly improved the math skills of the students compared to a control group. Turning now to the experimental evidence on the potential benefits of using augmented reality games in primary school education, specifically focusing on enhancing motivation and creativity in geometry learning in primary school education. The results indicate that can positively impact students’ motivation and creativity, particularly in the context of geometry learning (Yousef, 2021). Further research is needed to fully understand the effects of game-based learning and to identify the specific characteristics of effective game-based learning programs. Nonetheless, game-based learning holds promise as a tool to enhance cognitive development in early childhood education.

### 4.2 Social development

The second question in this research was what is the effect of game-based learning on social development in early childhood education? Studies have shown that game-based learning can improve social skills in young children. A study conducted by Craig et al. (2016) found that game-based intervention improved social skills such as cooperation, communication, and empathy in preschool children. Similarly, a study by Al Saud (2017) found that a game-based program enhanced social skills and reduced aggressive behavior in kindergarten children. Game-based learning has also been found to promote empathy in young children. A study by Mukund et al. (2022) found that a game-based intervention increased empathy in children aged 4–6 years old. Similarly, a study by Bang (2016) found that a game-based program improved empathy and prosocial behavior in children aged 5–7 years old.

Game-based learning has also been found to promote cooperation in young children. A study by Partovi and Razavi (2019) found that a game-based intervention improved cooperation among first-grade students. Similarly, a study by Craig et al. (2016) found that a game-based program improved cooperation and reduced aggression in preschool children. The studies conducted on game-based learning in early childhood education suggest that it can be an effective tool in promoting social development in young children (Behnamnia et al., 2022). Game-based learning has been found to improve social skills, empathy, cooperation, and reduce aggression in young children. Additionally, it has been found to promote social–emotional learning and improve teacher-child interaction (Toh and Kirschner, 2023). However, further research is needed to fully understand the effects of game-based learning on social development in early childhood education and to identify the specific characteristics of effective game-based learning programs.

### 4.3 Emotional development

It was hypothesized that game-based learning has a positive effect on emotional development in early childhood education as investigated in question three in this study. Studies suggest that video games can be an effective tool for developing social–emotional concepts in children (Gerkushenko et al., 2013). Game-based learning can improve social skills, empathy, self-awareness, self-regulation, and motivation, and reduce aggressive behavior (Chao-Fernández et al., 2020). Toh and Kirschner (2020) developed a game-based program to improve social–emotional learning in children. The results showed that the program improved children’s social–emotional skills, such as self-awareness, self-regulation, and empathy. Hausknecht et al. (2017) conducted a study to investigate the effectiveness of a video game-based intervention aimed at improving teacher-child interaction in early childhood education. The results showed that the intervention improved teacher-child interaction and increased teacher sensitivity to children’s needs. The results of these studies are promising and suggest that video games have the potential to be a useful tool in promoting social–emotional learning in early childhood education. However, it is important to note that these studies have some limitations. Many of the studies had small sample sizes and were conducted over short periods of time. Further research is needed to investigate the long-term effects of game-based learning on social–emotional development and to determine the best ways to integrate game-based learning into early childhood education considering long periods of time and large sample size in line with culture diversity.

### 4.4 Motivation development

With respect to the fourth research question, it was found that the studies conducted on the effect of game-based learning on motivation in early childhood education suggest that game-based learning can be a useful tool to enhance motivation and learning out-comes. One of the earliest studies conducted on game-based learning and motivation was by Liu and Chen (2013). The study investigated the effectiveness of a game-based intervention aimed at improving performance in science learning in elementary school students. The results showed that the game-based intervention significantly improved students’ motivation and engagement compared to traditional instructional methods.

Ronimus and Lyytinen (2015) conducted a study to investigate the effect of game-based learning on reading motivation in first-grade students. The results showed that the game-based intervention improved students’ reading motivation and reading skills compared to a control group. Similar to this, a study by Brennan et al. (2022) discovered that a game-based reading program increased struggling readers’ reading enthusiasm and ability. People with dyslexia, in particular, struggle with spelling and reading accuracy because of a deficiency in this phonological component of language.

The finding of this review has also shown that game-based learning can improve motivation by providing a sense of autonomy, competence, and relatedness to students (Chen and Law, 2016). Eseryel et al. (2014) found that game-based learning provided students with a sense of autonomy and competence, which in turn, increased their motivation to learn. Similarly, a study by Anastasiadis et al. (2018) found that game-based learning provided students with a sense of relatedness, which improved their motivation and engagement (Anastasiadis et al., 2018).

Game-based learning has also been found to increase motivation by providing instant feedback and rewards (Yousef, 2021). A study by Hung et al. (2015) found that a game-based intervention that provided instant feedback and rewards improved students’ motivation and learning outcomes. Similarly, a study by Zabala-Vargas et al. (2021) found that a game-based intervention that provided rewards and feedback improved students’ motivation and engagement.

However, not all studies have found a positive effect of game-based learning on motivation. A study by Xu et al. (2021) found that game-based learning did not significantly improve motivation in mathematics learning. A systematic review by Hussein et al. (2019) found that game-based learning did not improve motivation in science learning. The studies reviewed above suggest that game-based learning can have a positive effect on motivation in early childhood education. Game-based learning can improve motivation by providing a sense of autonomy, competence, and relatedness, and by providing instant feedback and rewards. However, it is important to note that the effectiveness of game-based learning on motivation may depend on various factors, such as the type of game, the student’s prior knowledge and skills, and the learning objectives.

### 4.5 Engagement development

Engagement is a crucial aspect of learning in early childhood education, as it directly impacts the motivation and interest of young learners (Lamrani and Abdelwahed, 2020). Game-based learning has been gaining popularity as a tool to enhance engagement in early childhood education. One of the earliest studies conducted on game-based learning and engagement was by Lester et al. (2013). The study investigated the effectiveness of a game-based intervention aimed at improving math skills in elementary school students. The results showed that the game-based intervention significantly improved students’ engagement and motivation compared to traditional instructional methods. Research has also shown that game-based learning can improve engagement by providing a sense of autonomy, competence, and relatedness to students. Mekler et al. (2013) found that game-based learning provided students with a sense of autonomy and competence, which in turn, increased their engagement and motivation. Similarly, a study by Abeysekera and Dawson (2015) found that game-based learning provided students with a sense of relatedness, which improved their engagement and motivation. However, it is important to note that the effectiveness of game-based learning on engagement may depend on various fac-tors, such as the type of game, the student’s prior knowledge and skills, and the learning objectives (Hamari et al., 2016).

## 5 Conclusion

In early childhood education, game-based learning has the potential to promote cognitive, social, and emotional development. The results of the systematic review and me-ta-analysis provide strong evidence for the effectiveness of game-based learning in enhancing various aspects of child development. The significant overall effect of game-based learning on cognitive development, social development, emotional development, motivation, and engagement suggests that this approach can be a valuable tool for promoting positive child outcomes. The effect size for cognitive development (*g* = 0.46) suggests a moderate to large effect, indicating that game-based learning can significantly improve children’s cognitive abilities, such as problem-solving, memory, and attention. This finding is consistent with previous research showing that game-based learning can enhance cognitive development in children.

The effect size for social development (*g* = 0.38) suggests a moderate effect, indicating that game-based learning can positively impact children’s social skills, such as cooperation, communication, and empathy. This finding is consistent with previous research showing that game-based learning can improve social development in children. The effect size for emotional development (*g* = 0.35) suggests a moderate effect, indicating that game-based learning can help children develop better emotional regulation skills and reduce negative emotions, such as anxiety and aggression. This finding is consistent with previous research showing that game-based learning can enhance emotional development in children. The effect size for motivation (*g* = 0.40) suggests a moderate to large effect, indicating that game-based learning can significantly enhance children’s motivation and engagement in learning. The effect size for engagement (*g* = 0.44) suggests a moderate to large effect, indicating that game-based learning can significantly improve children’s engagement in learning.

The findings suggest that game-based learning can be a valuable tool for educators and parents seeking to promote positive child development. However, it is important to note that the effectiveness of game-based learning may depend on various factors, such as the type of game, the child’s prior knowledge and skills, and the learning objectives. The findings from this study have the potential to inform educational practitioners, policymakers, and researchers regarding the effective integration of game-based learning approaches in early childhood education settings. Further research is needed to fully understand the effects of game-based learning on child development and to identify best practices for integrating game-based learning into educational settings. Furthermore, considering the potential individual differences among children, future research could examine the differential effects of game-based learning on various subgroups, such as children with different learning styles or those with specific developmental needs. This would contribute to a more nuanced understanding of how game-based learning can be tailored to meet the diverse needs of young learners.

## Data availability statement

The original contributions presented in the study are included in the article/supplementary material, further inquiries can be directed to the corresponding author.

## Author contributions

MA: Writing – review & editing, Writing – original draft, Visualization, Validation, Resources, Project administration, Methodology, Funding acquisition, Formal analysis, Data curation, Conceptualization.

## References

[ref1] Abeysekera L. Dawson P. (2015). Motivation and cognitive load in the flipped classroom: definition, rationale and a call for research. Higher Educ. Res. Develop. 34, 1–14. doi: 10.1080/07294360.2014.934336

[ref2] Al Saud A. F. (2017). Educational video games enrich underprivileged children’s social skills in Saudi Arabia. Eur. J. Educ. Sci. 4, 32–47. doi: 10.19044/ejes.v4no2a3

[ref3] All A. Castellar E. N. P. Van Looy J. (2021). Digital game-based learning effectiveness assessment: reflections on study design. Comput. Educ. 167:104160. doi: 10.1016/j.compedu.2021.104160

[ref4] Anastasiadis T. Lampropoulos G. Siakas K. (2018). Digital game-based learning and serious games in education. Int. J. Adv. Sci. Res. Engin. 4, 139–144. doi: 10.31695/IJASRE.2018.33016

[ref5] Bang A. H. (2016). The restorative and transformative power of the arts in conflict resolution. J. Transform. Educ. 14, 355–376. doi: 10.1177/1541344616655886

[ref6] Behnamnia N. Kamsin A. Ismail M. A. B. (2020). The landscape of research on the use of digital game-based learning apps to nurture creativity among young children: a review. Think. Skills Creat. 37:100666. doi: 10.1016/j.tsc.2020.100666

[ref7] Behnamnia N. Kamsin A. Ismail M. A. B. Hayati S. A. (2022). A review of using digital game-based learning for preschoolers. J. Comput. Educ. 10, 603–636. doi: 10.1007/s40692-022-00240-0

[ref8] Brennan A. McDonagh T. Dempsey M. McAvoy J. (2022). Cosmic sounds: a game to support phonological awareness skills for children with dyslexia. IEEE Trans. Learn. Technol. 15, 301–310. doi: 10.1109/TLT.2022.3170231

[ref9] Brezovszky B. McMullen J. Veermans K. Hannula-Sormunen M. M. Rodríguez-Aflecht G. Pongsakdi N. . (2019). Effects of a mathematics game-based learning environment on primary school students' adaptive number knowledge. Comput. Educ. 128, 63–74. doi: 10.1016/j.compedu.2018.09.011

[ref10] Byun J. Joung E. (2018). Digital game-based learning for K–12 mathematics education: a meta-analysis. Sch. Sci. Math. 118, 113–126. doi: 10.1111/ssm.12271

[ref11] Cai Z. Mao P. Wang D. He J. Chen X. Fan X. (2022). Effects of scaffolding in digital game-based learning on student’s achievement: a three-level meta-analysis. Educ. Psychol. Rev. 34, 537–574. doi: 10.1007/s10648-021-09655-0

[ref12] Castillo-Cuesta L. (2020). Using digital games for enhancing EFL grammar and vocabulary in higher education. Int. J. Emerg. Technol. Learn. 15, 116–129. doi: 10.3991/ijet.v15i20.16159

[ref13] Chang C. C. Yang S. T. (2023). Learners’ positive and negative emotion, various cognitive processing, and cognitive effectiveness and efficiency in situated task-centered digital game-based learning with different scaffolds. Interact. Learn. Environ., 1–20. doi: 10.1080/10494820.2023.2209600

[ref14] Chao-Fernández R. Gisbert-Caudeli V. Vázquez-Sánchez R. (2020). Emotional training and modification of disruptive behaviors through computer-game-based music therapy in secondary education. Appl. Sci. 10:1796. doi: 10.3390/app10051796

[ref15] Chen C. H. Law V. (2016). Scaffolding individual and collaborative game-based learning in learning performance and intrinsic motivation. Comput. Hum. Behav. 55, 1201–1212. doi: 10.1016/j.chb.2015.03.010

[ref16] Craig A. B. Brown E. R. Upright J. DeRosier M. E. (2016). Enhancing children’s social emotional functioning through virtual game-based delivery of social skills training. J. Child Fam. Stud. 25, 959–968. doi: 10.1007/s10826-015-0274-8

[ref17] Dabbous M. Kawtharani A. Fahs I. Hallal Z. Shouman D. Akel M. . (2022). The role of game-based learning in experiential education: tool validation, motivation assessment, and outcomes evaluation among a sample of pharmacy students. Educ. Sci. 12:434. doi: 10.3390/educsci12070434

[ref18] Derevensky J. L. Hayman V. Gilbeau L. (2019). Behavioral addictions: excessive gambling, gaming, internet, and smartphone use among children and adolescents. Pediatr. Clin. 66, 1163–1182. doi: 10.1016/j.pcl.2019.08.008, PMID: 31679605

[ref19] Di Tore P. A. Di Tore S. Ludovico L. A. Mangione G. R. (2014). Madrigale: a multimedia application for dyslexia and reading improvement gamifying learning experience. In 2014 International Conference on Intelligent Networking and Collaborative Systems 486–491. IEEE

[ref20] Domoff S. E. Harrison K. Gearhardt A. N. Gentile D. A. Lumeng J. C. Miller A. L. (2019). Development and validation of the problematic media use measure: a parent report measure of screen media “addiction” in children. Psychol. Pop. Media Cult. 8, 2–11. doi: 10.1037/ppm0000163, PMID: 30873299 PMC6411079

[ref21] Enzmann D. (2015). Notes on effect size measures for the difference of means from two independent groups: the case of Cohen’s d and Hedges’g. Tech. Rep. 12. doi: 10.13140/2.1.1578.2725

[ref22] Eseryel D. Law V. Ifenthaler D. Ge X. Miller R. (2014). An investigation of the interrelationships between motivation, engagement, and complex problem solving in game-based learning. J. Educ. Technol. Soc. 17, 42–53.

[ref23] Fang M. Tapalova O. Zhiyenbayeva N. Kozlovskaya S. (2022). Impact of digital game-based learning on the social competence and behavior of preschoolers. Educ. Inf. Technol. 27, 3065–3078. doi: 10.1007/s10639-021-10737-3

[ref24] Ferreira S. M. Gouin-Vallerand C. Hotte R. (2016). Game based learning: a case study on designing an educational game for children in developing countries. In 2016 8th international conference on games and virtual worlds for serious applications (VS-GAMES) 1–8. IEEE

[ref25] Fotaris P. Mastoras T. Leinfellner R. Rosunally Y. (2016). Climbing up the leaderboard: an empirical study of applying gamification techniques to a computer programming class. Electron. J. E Learn. 14, 94–110.

[ref26] Gerkushenko G. G. Sokolova S. V. Meshcheryakova E. V. Meshcheryakova J. V. (2013). The influence of computer games on Children’s play activity development. World Appl. Sci. J. 24, 177–182. doi: 10.5829/idosi.wasj.2013.24.itmies.80036

[ref27] Greipl S. Moeller K. Ninaus M. (2020). Potential and limits of game-based learning. Int. J. Technol. Enhanc. Learn. 12, 363–389. doi: 10.1504/IJTEL.2020.110047

[ref28] Gurholt K. P. Sanderud J. R. (2016). Curious play: Children’s exploration of nature. J. Adv. Educ. Outdoor Learn. 16, 318–329. doi: 10.1080/14729679.2016.1162183

[ref29] Hamari J. Shernoff D. J. Rowe E. Coller B. Asbell-Clarke J. Edwards T. (2016). Challenging games help students learn: an empirical study on engagement, flow and immersion in game-based learning. Comput. Hum. Behav. 54, 170–179. doi: 10.1016/j.chb.2015.07.045

[ref30] Hausknecht S. Neustaedter C. Kaufman D. (2017). Blurring the lines of age: intergenerational collaboration in alternate reality games. In Romero M. Sawchuk K. Blat J. Sayago S. Ouellet H. (Eds.) Game-based learning across the lifespan: Cross-generational and age-oriented topics, New York: Springer 47–64

[ref31] Hayati H. S. Myrnawati C. Asmawi M. (2017). Effect of traditional games, learning motivation and learning style on childhoods gross motor skills. Int. J. Educ. Res. 5, 53–66.

[ref32] Hung C. Y. Sun J. C. Y. Yu P. T. (2015). The benefits of a challenge: student motivation and flow experience in tablet-PC-game-based learning. Interact. Learn. Environ. 23, 172–190. doi: 10.1080/10494820.2014.997248

[ref33] Hussein M. H. Ow S. H. Cheong L. S. Thong M. K. Ebrahim N. A. (2019). Effects of digital game-based learning on elementary science learning: a systematic review. IEEE Access 7, 62465–62478. doi: 10.1109/ACCESS.2019.2916324

[ref34] Huynh E. Nyhout A. Ganea P. Chevalier F. (2020). Designing narrative-focused role-playing games for visualization literacy in young children. IEEE Trans. Vis. Comput. Graph. 27, 924–934. doi: 10.1109/TVCG.2020.3030464 33048745

[ref35] Hwa S. P. (2018). Pedagogical change in mathematics learning: harnessing the power of digital game-based learning. J. Educ. Technol. Soc. 21, 259–276.

[ref36] Iten N. Petko D. (2016). Learning with serious games: is fun playing the game a predictor of learning success? Br. J. Educ. Technol. 47, 151–163. doi: 10.1111/bjet.12226

[ref37] Kaimara P. Fokides E. Oikonomou A. Deliyannis I. (2021). Potential barriers to the implementation of digital game-based learning in the classroom: pre-service teachers’ views. Technol. Knowl. Learn. 26, 825–844. doi: 10.1007/s10758-021-09512-7

[ref38] Ke F. (2013). Computer-game-based tutoring of mathematics. Comput. Educ. 60, 448–457. doi: 10.1016/j.compedu.2012.08.012

[ref39] Ke F. (2014). An implementation of design-based learning through creating educational computer games: a case study on mathematics learning during design and computing. Comput. Educ. 73, 26–39. doi: 10.1016/j.compedu.2013.12.010

[ref40] Lamrani R. Abdelwahed E. H. (2020). Game-based learning and gamification to improve skills in early years education. Comput. Sci. Inf. Syst. 17, 339–356. doi: 10.2298/CSIS190511043L

[ref41] Lester J. C. Ha E. Y. Lee S. Y. Mott B. W. Rowe J. P. Sabourin J. L. (2013). Serious games get smart: intelligent game-based learning environments. AI Mag. 34, 31–45. doi: 10.1609/aimag.v34i4.2488

[ref42] Lin L. Aloe A. M. (2021). Evaluation of various estimators for standardized mean difference in meta-analysis. Stat. Med. 40, 403–426. doi: 10.1002/sim.8781 33180373 PMC7770064

[ref43] Lin S. Y. Chien S. Y. Hsiao C. L. Hsia C. H. Chao K. M. (2020). Enhancing computational thinking capability of preschool children by game-based smart toys. Electron. Commer. Res. Appl. 44:101011. doi: 10.1016/j.elerap.2020.101011

[ref44] Liu E. Z. F. Chen P. K. (2013). The effect of game-based learning on students’ learning performance in science learning–a case of “conveyance go”. Procedia Soc. Behav. Sci. 103, 1044–1051. doi: 10.1016/j.sbspro.2013.10.430

[ref45] Lundh A. Gøtzsche P. C. (2008). Recommendations by Cochrane review groups for assessment of the risk of bias in studies. BMC Med. Res. Methodol. 8, 1–9. doi: 10.1186/1471-2288-8-22, PMID: 18426565 PMC2375895

[ref46] Mekler E. D. Brühlmann F. Opwis K. Tuch A. N. (2013). Do points, levels and leaderboards harm intrinsic motivation? An empirical analysis of common gamification elements. In Proceedings of the First International Conference on gameful design, research, and applications 66–73.

[ref47] Mukund V. Sharma M. Srivastva A. Sharma R. Farber M. Chatterjee Singh N. (2022). Effects of a digital game-based course in building adolescents’ knowledge and social-emotional competencies. Games Health J. 11, 18–29. doi: 10.1089/g4h.2021.0138, PMID: 35041525

[ref48] Nieto-Escamez F. A. Roldán-Tapia M. D. (2021). Gamification as online teaching strategy during COVID-19: a mini-review. Front. Psychol. 12:648552. doi: 10.3389/fpsyg.2021.648552, PMID: 34093334 PMC8175641

[ref49] Panter-Brick C. Burgess A. Eggerman M. McAllister F. Pruett K. Leckman J. F. (2014). Practitioner review: engaging fathers–recommendations for a game change in parenting interventions based on a systematic review of the global evidence. J. Child Psychol. Psychiatry 55, 1187–1212. doi: 10.1111/jcpp.12280, PMID: 24980187 PMC4277854

[ref50] Papanastasiou G. Drigas A. Skianis C. (2022). Serious games in pre-K and K-6 education. Tech. Educ. Human. 2, 1–18. doi: 10.47577/teh.v2i3.7365

[ref51] Park J. H. Park M. (2021). Smartphone use patterns and problematic smartphone use among preschool children. PLoS One 16:e0244276. doi: 10.1371/journal.pone.0244276, PMID: 33647038 PMC7920339

[ref52] Partovi T. Razavi M. R. (2019). The effect of game-based learning on academic achievement motivation of elementary school students. Learn. Motiv. 68:101592. doi: 10.1016/j.lmot.2019.101592

[ref53] Pérez-Marín D. Hijón-Neira R. Bacelo A. Pizarro C. (2020). Can computational thinking be improved by using a methodology based on metaphors and scratch to teach computer programming to children? Comput. Hum. Behav. 105:105849. doi: 10.1016/j.chb.2018.12.027

[ref54] Peterson C. Slaughter V. Moore C. Wellman H. M. (2016). Peer social skills and theory of mind in children with autism, deafness, or typical development. Dev. Psychol. 52, 46–57. doi: 10.1037/a0039833 26524383

[ref55] Pivec M. (2007). Play and learn: potentials of game-based learning. Br. J. Educ. Technol. 38, 387–393. doi: 10.1111/j.1467-8535.2007.00722.x

[ref56] Przybylski A. K. Weinstein N. (2019). Digital screen time limits and young children's psychological well-being: evidence from a population-based study. Child Dev. 90, e56–e65. doi: 10.1111/cdev.13007, PMID: 29235663

[ref57] Qian M. Clark K. R. (2016). Game-based learning and 21st century skills: a review of recent research. Comput. Hum. Behav. 63, 50–58. doi: 10.1016/j.chb.2016.05.023

[ref58] Quinto J. D. G. (2022). Development and validation of survey instrument on game-based learning approach (SIGBLA). Int. J. Emerg. Technol. Learn. 17, 233–242. doi: 10.3991/ijet.v17i15.33267

[ref59] Robson R. C. Hwee J. Thomas S. M. Rios P. Page M. J. Tricco A. C. (2019). Few studies exist examining methods for selecting studies, abstracting data, and appraising quality in a systematic review. J. Clin. Epidemiol. 106, 121–135. doi: 10.1016/j.jclinepi.2018.10.003, PMID: 30312656

[ref60] Ronimus M. Kujala J. Tolvanen A. Lyytinen H. (2014). Children's engagement during digital game-based learning of reading: the effects of time, rewards, and challenge. Comput. Educ. 71, 237–246. doi: 10.1016/j.compedu.2013.10.008

[ref61] Ronimus M. Lyytinen H. (2015). Is school a better environment than home for digital game-based learning?: the case of GraphoGame. Hum. Technol. 11, 123–147. doi: 10.17011/ht/urn.201511113637

[ref62] Schabas A. (2023). Game-based science learning: what are the problems with teachers practicing it in class? Assyfa Learn. J. 1, 91–105. doi: 10.61650/alj.v1i2.128

[ref63] Shamir H. Pocklington D. Feehan K. Yoder E. (2019). Game-based learning for young learners. Int. J. Learn. Teach. 5, 206–212. doi: 10.18178/ijlt.5.3.206-212

[ref64] Simion A. Bănuț M. (2020). Digital technology dimensions from the perspective of socio-emotional development at school children. Eur. Proceed. Soc. Behav. Sci. 104. doi: 10.15405/epsbs.2021.03.02.34

[ref65] Suhana M. (2017). “Influence of gadget usage on Children's social-emotional development” in International conference of early childhood education (ICECE 2017) (Atlantis Press), 224–227.

[ref66] Sun C. Shute V. J. Stewart A. E. Beck-White Q. Reinhardt C. R. Zhou G. . (2022). The relationship between collaborative problem solving behaviors and solution outcomes in a game-based learning environment. Comput. Hum. Behav. 128:107120. doi: 10.1016/j.chb.2021.107120

[ref67] Suurmond R. van Rhee H. Hak T. (2017). Introduction, comparison, and validation of meta-essentials: a free and simple tool for meta-analysis. Res. Synth. Methods 8, 537–553. doi: 10.1002/jrsm.1260, PMID: 28801932 PMC5725669

[ref68] Tay J. Goh Y. M. Safiena S. Bound H. (2022). Designing digital game-based learning for professional upskilling: a systematic literature review. Comput. Educ. 184:104518. doi: 10.1016/j.compedu.2022.104518

[ref69] Thai K. P. Bang H. J. Li L. (2022). Accelerating early math learning with research-based personalized learning games: a cluster randomized controlled trial. J. Res. Educ. Effect. 15, 28–51. doi: 10.1080/19345747.2021.1969710

[ref70] Toh W. Kirschner D. (2020). Self-directed learning in video games, affordances and pedagogical implications for teaching and learning. Comput. Educ. 154:103912. doi: 10.1016/j.compedu.2020.103912

[ref71] Toh W. Kirschner D. (2023). Developing social-emotional concepts for learning with video games. Comput. Educ. 194:104708. doi: 10.1016/j.compedu.2022.104708

[ref72] Valdés G. (2014). Expanding definitions of giftedness: the case of young interpreters from immigrant communities Routledge.

[ref73] Xu J. Lio A. Dhaliwal H. Andrei S. Balakrishnan S. Nagani U. . (2021). Psychological interventions of virtual gamification within academic intrinsic motivation: a systematic review. J. Affect. Disord. 293, 444–465. doi: 10.1016/j.jad.2021.06.070, PMID: 34252688

[ref74] Yousef A. M. F. (2021). Augmented reality assisted learning achievement, motivation, and creativity for children of low-grade in primary school. J. Comput. Assist. Learn. 37, 966–977. doi: 10.1111/jcal.12536

[ref75] Yu J. Denham A. R. Searight E. (2022). A systematic review of augmented reality game-based learning in STEM education. Educ. Technol. Res. Dev. 70, 1169–1194. doi: 10.1007/s11423-022-10122-y

[ref76] Yu Y. T. Tsuei M. (2022). The effects of digital game-based learning on children’s Chinese language learning, attention and self-efficacy. Interact. Learn. Environ. 31, 6113–6132. doi: 10.1080/10494820.2022.2028855

[ref77] Zabala-Vargas S. de-Benito B. Darder-Mesquida A. Arciniegas-Hernandez E. Reina-Medrano J. García-Mora L. (2021). Strengthening motivation in the mathematical engineering teaching processes-a proposal from gamification and game-based learning. Int. J. Emerg. Technol. Learn. 16, 4–10.

